# Intracellular injection of phospholipids directly alters exocytosis and the fraction of chemical release in chromaffin cells as measured by nano-electrochemistry†

**DOI:** 10.1039/d0sc03683h

**Published:** 2020-10-06

**Authors:** Mohaddeseh Aref, Elias Ranjbari, Armaghan Romiani, Andrew G. Ewing

**Affiliations:** Department of Chemistry and Molecular Biology, University of Gothenburg Gothenburg Sweden andrew.ewing@chem.gu.se

## Abstract

Using a nano-injection method, we introduced phospholipids having different intrinsic geometries into single secretory cells and used single cell amperometry (SCA) and intracellular vesicle impact electrochemical cytometry (IVIEC) with nanotip electrodes to monitor the effects of intracellular incubation on the exocytosis process and vesicular storage. Combining tools, this work provides new information to understand the impact of intracellular membrane lipid engineering on exocytotic release, vesicular content and fraction of chemical release. We also assessed the effect of membrane lipid alteration on catecholamine storage of isolated vesicles by implementing another amperometric technique, vesicle impact electrochemical cytometry (VIEC), outside the cell. Exocytosis analysis reveals that the intracellular nano-injection of phosphatidylcholine and lysophosphatidylcholine decreases the number of released catecholamines, whereas phosphatidylethanolamine shows the opposite effect. These observations support the emerging hypothesis that lipid curvature results in membrane remodeling through secretory pathways, and also provide new evidence for a critical role of the lipid localization in modulating the release process. Interestingly, the IVIEC data imply that total vesicular content is also affected by *in situ* supplementation of the cells with some lipids, while, the corresponding VIEC results show that the neurotransmitter content in isolated vesicles is not affected by altering the vesicle membrane lipids. This suggests that the intervention of phospholipids inside the cell has its effect on the cellular machinery for vesicle release rather than vesicle structure, and leads to the somewhat surprising conclusion that modulating release has a direct effect on vesicle structure, which is likely due to the vesicles opening and closing again during exocytosis. These findings could lead to a novel regulatory mechanism for the exocytotic or synaptic strength based on lipid heterogeneity across the cell membrane.

## Abstract 2

Amperometry and intracellular vesicle impact electrochemical cytometry with nanotip electrodes were used to monitor the effects on exocytosis and vesicular storage after nano-injection of phospholipids with different geometries into secretory cells.

## Graphical abstract

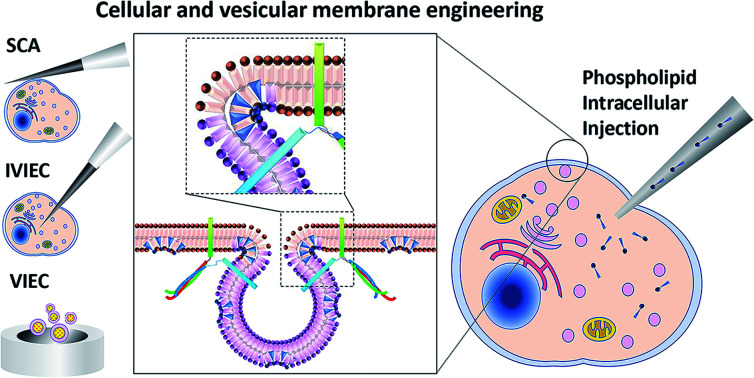

## Introduction

Membrane lipids undoubtedly appear to contribute to almost every step in the regulated secretory pathway from the biogenesis of secretory granules to the exocytosis process.^1^ Regulated exocytosis, a crucial process for cell-to-cell communication, is a calcium-dependent mechanism that entails the fusion of a secretory organelle with cellular plasma membrane followed by the partial release of its content.^2,3^ This complex release process can be postulated as a molecular mechanism for altering synaptic strength and hence is likely associated with phenomena like learning and memory.^4^ The molecular machinery underlying regulated exocytosis involves interplay between the membrane proteins and phospholipids.^5^ In addition to the important function of proteinaceous players in the secretory journey of granules, phospholipids also contribute to the key steps.

Cellular and vesicular membranes are composed of a broad spectrum of phospholipids with specific properties that can directly influence membrane topology, dynamics, and tasks.^1^ Phospholipids are localized in the two leaflets of the cell membrane, asymmetrically. For instance, the majority of phosphatidylethanolamine (PE) preferentially localize to the inner leaflet of the cell membrane, while phosphatidylcholine (PC) segregates to the outer leaflet. There is no doubt that an alteration of the subtle cellular and vesicular lipid balance can cause the alteration in the exocytosis process.^6,7^ Kato *et al.* observed an inhibited exocytosis process in the cells with homogeneous membranes revealing that the unequal distribution of lipids with respect to the inner and outer leaflets of the cell membrane is vital for cell survival.^8^ However, probing the influence of lipid changes on the membrane dynamics during the secretory pathway requires an analytical method to directly measure neurotransmission strength at the single cell level.

Single-cell amperometry (SCA) is the most used analytical technique to study quantitatively the individual exocytosis events at the single-cell level, allowing quantification of secreted electroactive neurotransmitters.^9–11^ Moreover, the high temporal resolution of SCA provides important information about the dynamics of the fusion pore formed during the exocytosis process. Intracellular vesicle impact electrochemical cytometry (IVIEC), a method developed in the Ewing group, is another analytical technique, in this case offering quantification of vesicular neurotransmitter content inside the cytoplasm of individual living cells using a conical nanotip electrode.^12,13^ The fraction of catecholamines secreted during exocytosis at the single-cell level can be calculated by combining the results of these two techniques.^10^ Recently, combination of SCA and IVIEC at carbon nanotip electrodes has been successfully applied to study the effect of several pharmacological and chemical treatments on single cell exocytosis including investigation of the effects of extracellular ATP on exocytosis of bovine chromaffin cells,^11^ and to study the influence of the cognition altering drugs (cocaine and methylphenidate) on exocytotic release and vesicle content in pheochromocytoma (PC12) cells.^10^ Also, a very recent study was done by combining these two techniques to reveal the possible link between the fraction of neurotransmitter release, synaptic strength and plasticity.^14^ Vesicle impact electrochemical cytometry (VIEC) is similar to IVIEC, but instead of *in situ* quantification of vesicle content, vesicles are isolated and maintained as a suspension in an intracellular physiological buffer, and then the electrochemical cytometry is performed on the isolated vesicles.^15^ In this way, it is possible to chemically or pharmacologically treat vesicles without interference from the cellular machinery.

The amount of neurotransmitter released per exocytosis event can be altered by influencing the lipid composition of the plasma membrane. The most direct evidence for this alteration was presented by both the Ewing and Amatore groups using amperometric methods after incubation of model cells with different types of phospholipids.^6,7^ In both cases, the selected phospholipids were added to the cell culture medium outside the cell to study the effects of lipids on exocytosis. In addition, these experiments were either with short or long injections to try to modify only the outer membrane leaflet or the entire cell, respectively. At the time of those experiments it was not analytically feasible to restrict and isolate measurements to the inside of the cell, especially vesicular measurements.

In this paper, we have implemented SCA, IVIEC and VIEC to investigate the effects of intracellular lipids on exocytosis in bovine chromaffin cells and vesicles. The effect of cytoplasmic membrane engineering, lipid changes in the inner leaflet of the cell membrane and the outer leaflet of the vesicle membrane, on exocytosis and also vesicular content have been examined by combining these methods with a nano-injection method to deliver different phospholipids possessing different intrinsic geometries including cylindrical PC, conical PE, and inverse-conical lysophosphatidylcholine (LPC) into the single chromaffin cells using a nanopipette. The effects of this “intracellular incubation” of phospholipids on the exocytosis release process and the vesicular content were determined. Furthermore, we assessed whether the effect of intracellular incubation differs from the extracellular incubation on the membrane remodeling and how it could modulate the release process. Our results demonstrate that number of the molecules released during exocytosis and also the dynamics of exocytosis event from chromaffin single cells are altered by injection of the lipids into the individual cells; the *in situ* supplementation of PC and LPC decrease the number of released catecholamines, whereas PE shows the opposite effect. Interestingly, IVIEC data reveal that total vesicular content is also affected by *in situ* supplementation of the cells with some lipids, however, the corresponding VIEC results show that the neurotransmitter content from the isolated vesicle is not affected by altering the vesicle membrane lipids, suggesting that there is an intervention of phospholipids in the cellular machinery related to vesicle storage, but not in the isolated vesicles. These findings provide a key piece of data to the hypothesis that lipid heterogeneity and structure might be involved as a regulatory mechanism for exocytotic or synaptic strength in the initial stages of short-term memory.

## Results and discussion

### Intracellular supplementation with phospholipids regulates exocytotic release and dynamics

**Fig. 1:**
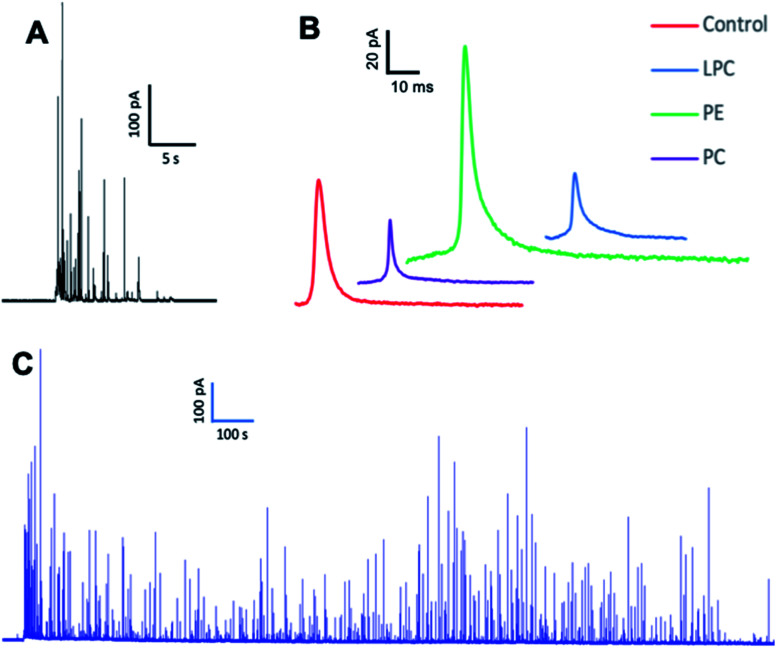
Representative amperometric traces obtained from (A) SCA, (B) the average current transients of SCA traces and (C) IVIEC on chromaffin cells with the nanotip carbon fiber electrodes after injection. The number of measured cells to obtain average current transients from SCA traces: control cells (*n* = 17) and cells *in situ* supplemented by PC (*n* = 15), PE (*n* = 18) and LPC (*n* = 15) from 3 unique chromaffin cell cultures. When possible, the same nanoelectrode was devoted to cell analysis to minimizes variability between measurements.

**Fig. 2:**
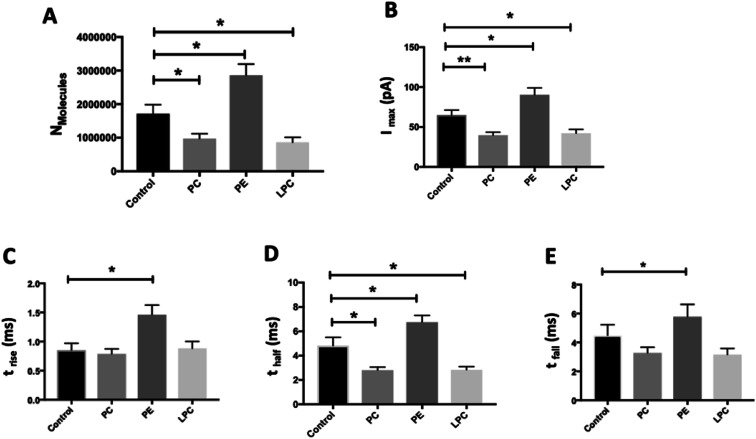
Summary of amperometric parameters from exocytosis for control and phospholipids-injected cells, including (A) *N*_molecules_, (B) *I*_max_, (C) *t*_rise_, (D) *t*_half_, and (E) *t*_fall_, after 30 min incubation by intracellular injection. The measurements were carried out with nanotip carbon fiber electrodes. Data are presented as means of medians and error bars are the standard error of the mean (SEM). Mann–Whitney two-tailed test. **: *p* < 0.01; *: *p* < 0.05. Number of the spikes analyzed: control cells (280 from 17 cells) and cells *in situ* supplemented by PC (351 from 15 cells), PE (246 from 18 cells) and LPC (409 from 15 cells) from 3 unique chromaffin cell cultures.

We employed SCA to monitor neurotransmitter release during exocytosis from control and phospholipid-treated bovine chromaffin cells. In this technique, a carbon fiber nanotip electrode as working electrode is placed on top of a cell under close observation by inverted microscopy and a reference electrode is positioned nearby in the surrounding buffered solution. Upon stimulation of the cells with a high-concentration K^+^ solution, vesicles docked at the cell membrane release part of the vesicular content. A constant applied potential between the working and reference electrodes leads to oxidation of electro-active molecules at the working electrode. Such events are observed in the form of current transients, which appear as spikes in a plot of current *versus* time (Fig. 1A and B). Integrating these spikes gives the total charge transferred (*Q*), which is related to the number of neurotransmitter molecules released (*N*) according to Faraday's law: *N* = *Q*/*nF*, where *n* is the number of electrons exchanged in the oxidation reaction (2e for catecholamines) and *F* is Faraday's constant (96 485 C mol^−1^). Although integration of the area under the current transient determines the number of released neurotransmitter molecules, the shape of the signal can be related to the release dynamics which provide certain information about the kinetics of the fusion pore. A typical SCA trace from the control cell is shown in Fig. 1A. Fig. 1B shows the average amperometric current transients obtained from SCA at control cells and at cells *in situ* supplemented by PC, PE, and LPC. For this experiment, 200 μM of PC, PE, and LPC were injected into the single cells separately and then after 30 min followed by stimulated exocytosis. The release parameters for the SCA results are shown in Fig. 2. The number of molecules released during exocytosis is altered when the inner leaflet of cell membrane and the outer leaflet of vesicle membrane are exposed to the different lipids by injection of the lipids into the individual cells (Fig. 2A). In comparison to the control cells, intracellular injection with PE increases the number of released neurotransmitters, whereas LPC and PC shows the opposite effect. To further understand the mechanism behind these variations, we investigated the effect from intracellular injection of phospholipids on the dynamics of the exocytotic process and fusion pore action by SCA peak analysis. The characteristics of individual spikes including *I*_max_, the peak amplitude, *t*_1/2_, the width of the SCA amperometric peak at its half amplitude, *t*_rise_, the time from 25% to 75% of the maximum amplitude at the rising part of the peak and *t*_fall_, the time from 75% to 25% of the maximum amplitude at the falling part of the peak were extracted using Igor Pro software.

**Scheme 1:**
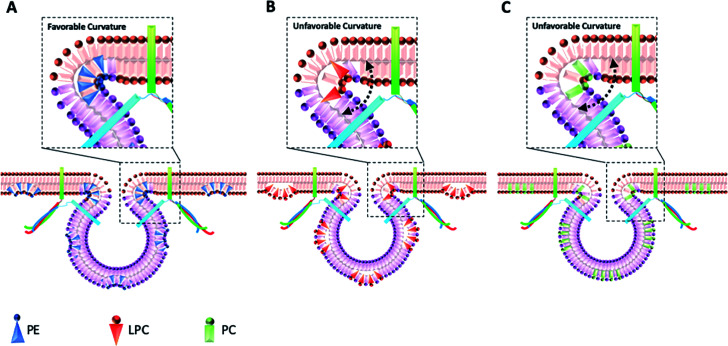
Schematic design for the proposed mechanism of cell membrane reconfiguration induced by injection of different phospholipids (A) PE, (B) LPC, and (C) PC into the cells. Injection of phospholipids mimics the endogenous lipids that change the balance of lipids ratio in the leaflets of cell and vesicle membranes.

The data in Fig. 2B show that intracellular injection of PE enhances *I*_max_ significantly, which indicates that catecholamine extrusion increases during exocytosis. Also, added PE appears to affect the time courses of exocytosis indicated by significantly slower peak rise (*t*_rise_), halfwidth (*t*_half_) and decay (*t*_fall_) times. The value of *t*_half_ corresponds to the duration of exocytotic events and is increased when PE added by intracellular injection. The observed variations comply with the stalk model represented for exocytosis by Ginsberg *et al.*^16^Scheme 1 visualizes the effect of lipid curvature on the feasibility of the pore formation. Among the selected lipids, PE is a conical shaped lipid, hence, its placement in the cytoplasmic membranes of the cell and vesicle induces the most favorable curvature for formation of a stable (longer lasting) fusion pore needed for exocytosis (Scheme 1A). This then leads to an elevated amount of neurotransmitter release. The values of *t*_rise_ and *t*_fall_ refer to the time of fusion pore opening and closing, respectively, and *t*_half_ is related to the time that the pore stays open. The data in Fig. 2C–E and Scheme 1A show that the pore opening time as well as its closing time tends to become longer which reflects the preferred curvature of the membrane in the presence of PE in the inner leaflet of the cell and the outer leaflet of the vesicle. The increase in *t*_rise_ is also due to this favorable curvature that helps widening the pore as much as possible.

The result for exocytosis of the cells which were supplemented with LPC and PC intracellularly showed that the number of molecules released from these cells decreased compared to untreated cells. LPC with its reverse cone shape (the opposite geometry *versus* PE) can induce a more strained membrane curvature during exocytosis (Scheme 1B, less stable fusion pore) and subsequently lead to a decrease in the pore opening duration (*t*_half_). Compared to control cells, the values of *t*_rise_ remained the same following intracellular LPC treatment; however, small, not statistically significant, decrease in *t*_fall_ is apparent which might cause the fusion pore to close slightly faster. In contrast, suppression in the *I*_max_ value was observed. The observed effects suggest that LPC might accelerate the dynamics of release such that in the presence of added LPC the pore stays open for a shorter time *versus* control. This subsequently results in a smaller number of molecules released during exocytosis event. Intracellular PC injection to affect the inner leaflet of the cell membrane and the outer leaflet of the vesicle membrane induces strained curvature in the pore due to the cylindrical geometry of the PC molecule (Scheme 1C), again making the pore unstable. This trend is similar to LPC, thus, allowing a smaller number of molecules to be released during exocytosis. Similar trends observed for PC and LPC on the dynamics of exocytosis might indicate that the specific enzymatic pathways that can inter-convert LPC and PC to each other are fast.^17^ While most studies have focused on the effects of extra-cellular lipids on the exocytosis,^6,7^ the results of this work provide a valuable perspective on the impact of the intracellular lipids on exocytotic release and dynamics. Table 1 compares the difference in the trends of previous works and present work on exocytotic parameters (see ESI for details explanation of Table 1†).

**Table 1:** Summary of the alterations in the exocytotic dynamics induced by intra- and extra-cellular phospholipids

Lipid	Shape	Added to	*N*	*I*_max_	*t*_rise_	*t*_half_	*t*_fall_	Reference
PE	Conical	Inner leaflet	↑	↑	↑	↑	↑	Present work
		Outer leaflet	—a	↑	—	↓	↓	Uchiyama *et al.*^6^
PC	Cylindrical	Inner leaflet	↓	↓	—	↓	—	Present work
		Outer leaflet	↓	↓	—	↑	↑	Uchiyama *et al.*^6^
LPC	Reverse-conical	Inner leaflet	↓	↓	—	↓	—	Present work
		Outer leaflet	↑	↑	↓	NR^b^	NR	Amatore *et al.*^7^

a Non-significant effect.

b Not reported.

Pre-spike feet from SCA were also analyzed to gain more information regarding the opening phase of the fusion pore during exocytosis. These pre-spike foot are recorded as small current transients prior to the main exocytotic peak, representing release of a small amount of neurotransmitter through a fusion pore formed at an early stage of vesicle fusion.^18^ The parameters including *I*_foot_, *t*_foot_, and *Q*_foot_ represent the duration of the foot, current amplitude, and charge of the pre-transient foot (associated with the number of neurotransmitters released through the foot),^19^ respectively, and are presented in Fig. S1.† The pre-spike feet characteristics of the exocytotic releases following intracellular exposure with PC, PE, and LPC showed slight but not significant alterations, suggesting that the of the initial fusion pore dynamics are not significantly affected.

### Effect of intracellular injection of phospholipids on vesicular content and fraction of release

**Fig. 3:**
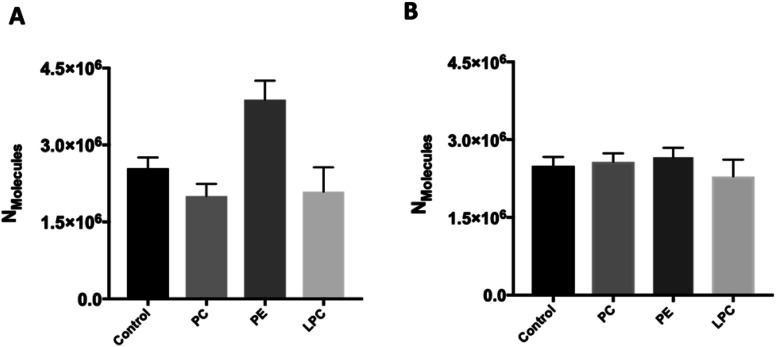
(A) The vesicular content as measured by IVIEC for the control and phospholipids-injected cells after 30 min incubation. The data shows no significant difference with 95% confidence interval, only the difference between control and PE was significant at 90% confidence interval. Number of the spikes analyzed: control cells (1407 from 12 cells) and cells *in situ* supplemented by PC (1489 from 14 cells), PE (1776 from 12 cells) and LPC (1506 from 12 cells) from 3 unique chromaffin cell cultures. (B) The vesicular content as measured by VIEC for the control and phospholipids-treated vesicles after 30 min incubation. Number of the spikes analyzed: control cells (2564 from 12 traces) and cells *in situ* supplemented by PC (2215 from 12 traces), PE (1883 from 10 traces) and LPC (1461 from 10 traces) from 3 unique chromaffin vesicle isolation. Data shows mean of median ± SEM. The data shows no significant difference with 95% confidence interval.

To investigate whether intracellular supplementation with PC, PE, and LPC causes an effect on vesicle content, IVIEC measurements were carried out. In IVIEC, a carbon fiber nanotip electrode is used to penetrate the cell membrane and quantify the neurotransmitter content of the single vesicles in the cytoplasm of individual living cells. In this method, the inserted electrode contacts the cell cytoplasm through which the vesicles diffuse. With a constant potential applied to the electrode, vesicles burst on the electrode surface. Here again, oxidation of the neurotransmitter content is observed as a current transient and the integration of these transients gives the total number of molecules inside of each individual vesicle that ruptured at the electrode surface. Fig. 1C illustrates an amperometric trace of transient currents (spikes) as a result of vesicles rupturing at the nanoelectrode surface inside a single-cell. The IVIEC data (Fig. 3A) depicts that PC and LPC treatment do not affect the vesicular content while interestingly there is a significant increase at 90% confidence interval (*p* value 0.0547) in the vesicular content after intracellular PE injection. The increased vesicle storage following *in situ* injection of PE might be interpreted as resulting from vesicular monoamine transporter-2 (VMAT-2) function. This transporter acts to pump catecholamines into the vesicle and is expressed in chromaffin granules of the adrenal medulla as the major transporter in bovine chromaffin vesicles.^20^ Although the regulatory mechanism of these transporters is still poorly understood, Kirshner *et al.* previously showed that the catecholamine uptake through the chromaffin granules VMAT-2 is ATP dependent.^21^ In addition, Tasseva *et al.* showed that PE contributes to mammalian mitochondrial function and a decrease in mitochondrial PE impairs cell growth, respiratory capacity, ATP production and profoundly alters mitochondrial morphology.^22^ Hence, enhancing the cytoplasmic PE concentration upon intracellular injection supplies higher contribution of this phospholipid with mitochondrial function and enhances the ATP production which can eventually increase the catecholamine storage of chromaffin vesicles, Scheme S1.†

We studied the effect of altering intracellular phospholipids on the fraction of neurotransmitters released during exocytosis by dividing the amount of release to the total vesicular content by combination of SCA and IVIEC. Partial release has been shown to be the dominant mode of exocytosis where only a fraction of the vesicle load is released during an event.^2,11^ Vesicular content and fraction released have been proposed to play an important role in regulation and plasticity in cellular communication.^2,12,23–25^ The fraction of catecholamine released was found to be 74%, 48%, 83%, and 41% for the control, and PC, PE, and LPC treated cells, respectively. These results show that fraction of release for PE is slightly increased compare to the control cells but it is decreased considerably after intracellular injection of either PC and LPC. To understand this observation, we need to consider the membrane remodeling presented in Scheme 1 again. According to the model in Scheme 1A, the slight alteration in the fraction of released after PE injection can be correlated to the favoring of the catecholamine release during exocytosis (number of molecules released and pore opening time, Fig. 2) and to the increase in the vesicular content obtained from IVIEC (Fig. 3A). Moreover, the remarkable variations in the fraction of release by LPC and PC again highlight the strained curvatures and disfavor exocytosis process induced by these lipids (Scheme 1B, C and Fig. 2), while the catecholamine content of vesicle did not alter significantly.

### Effect of phospholipid on the content of the isolated vesicles

The effect of the different phospholipids on isolated chromaffin vesicles was studied with VIEC outside the cell to determine if the changes following PE persists in the absence of the cellular machinery. We incubated solutions of isolated vesicles with the same concentration of the selected phospholipids and the same incubation time as the live cell experiment. VIEC measurement of vesicle content of isolated vesicles was carried out based on a previously procedure using a carbon fiber micro-disk electrode.^15^ Here, vesicles adsorb and rupture on an electrode kept at constant potential (700 mV), leading to rapid current transients. These events can be used to quantify the neurotransmitters stored inside isolated single vesicles. Perhaps surprisingly, the neurotransmitter content of the isolated vesicle did not change after incubation with PC, PE or LPC (Fig. 3B). The effect of PE on vesicular content observed with IVIEC after intracellular injection, but not with direct incubation of isolated vesicles in VIEC, suggests that cellular vesicles behave differently when exposed to the phospholipid and further suggest that PE is involved in the cellular machinery revolving around vesicle storage.

## Conclusions

In summary, in this work we developed intracellular lipid injection, as a promising strategy, to *in situ* manipulate the lipidic structure of not only the cell membrane inner leaflet but the outer leaflet of the vesicles. We then used this nano-injection method to add lipids with different intrinsic curvatures into single cells to investigate the impact of intracellular lipid composition on the exocytosis process of single cells and vesicle content. This work is the first demonstration of how changes in intracellular lipids can affect vesicular storage in a single cell. For this purpose, the IVIEC and VIEC techniques were used to discover the effect of lipids on vesicular content. Combining the SCA and IVIEC results, this work presents the fraction of catecholamine release affected by intracellular lipids. Our finding that intracellular lipid injection modulates exocytosis is in some ways comparable with the results for extracellular incubation of lipids by Amatore and coworkers in which they targeted only the composition of the outer cell membrane of bovine chromaffin cells with LPC and arachidonic acid, representing a cone shape similar to that of PE. They showed that when LPC is added to the outer leaflet of the cell membrane,^7^ it facilitates the neurotransmitter release together with larger amount of release, while inserting arachidonic acid to the outer cell membrane inhibits the exocytotic process and showed the smaller amount of release compare to control. Considering the intracellular supplementation of the membrane by LPC and PE in our model, the opposite effect of these phospholipids was observed in comparison to that of previous work^7^ where the outer membrane lipid composition was manipulated. This provides the novel, perhaps expected, finding that both the intrinsic membrane curvature for each phospholipid and the localization of the lipids in the membrane are important for membrane remodeling across the secretory pathway. This also has a crucial role on the fusion efficiency and subsequent effects on the release process and plasticity. Additionally, as phospholipids are the most abundant constituent of cellular membranes and it is accepted that an important part of drug action in the brain might be alteration of lipid distribution,^26^ our data could also be beneficial for development of drugs and novel pharmacological tools. As long-term impact, the combined approach of intracellular injection, SCA and IVIEC provides a sensitive analytical modality to understand how intracellular changes in lipid species, both in the inner membrane leaflet and the intracellular vesicles might be involved in relation to diseases involving memory and cognition.

## Experimental

### Nanotip carbon fiber electrode fabrication

Nanotip carbon fiber electrodes were fabricated as previously described.^13^ Briefly, A 5 μm diameter carbon fiber was aspirated into a borosilicate capillary (1.2 mm O.D., 0.69 mm I.D., Sutter Instrument Co., Novato, CA) and subsequently pulled in half. The carbon fiber outside the glass was cut to the length of 100–150 μm with a scalpel under a microscope and then flame etched by holding the electrode at the edge of a butane burner (Multiflame AB, Hässleholm, Sweden). The electrodes with the sharp and conical nanotip (about 100–200 nm in diameter, 30–100 μm in length) were subsequently sealed with epoxy (Epo-Tek, Billerica, MA) followed by a 10 s dipping in acetone to remove the carbon fiber from epoxy and finally cured at 100 °C in an oven overnight. Prior to experiments, electrodes were tested in a solution of 100 μM dopamine in PBS (pH 7.4) using cyclic voltammetry and only electrodes with diffusion limited steady-state current and low charging current were used.

### Nanopipette fabrication and injection setup

**Fig. 4:**
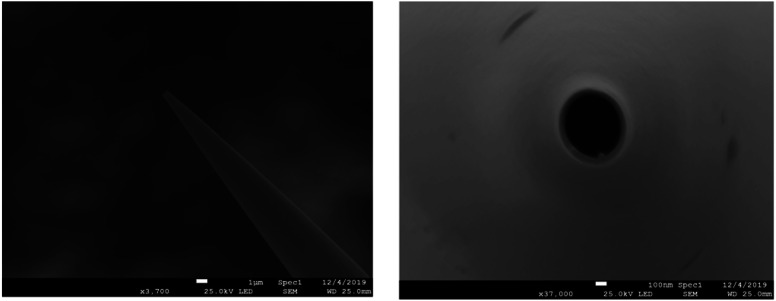
Scanning electron microscopy image of the fabricated nanopipette (side view and top view, from left to right).

Borosilicate capillaries (1.0 mm O.D.; 0.78 mm I.D.; 10 cm length, Sutter Instruments) were cleaned first by ethanol and then washed successively in acetone and pure water followed by 30 min sonication and then drying with nitrogen gas. The capillaries were pulled using a Sutter P-2000 laser puller (Sutter Instrument, Novato, CA). The aperture diameters of the nanopipettes obtained were ∼500 nm as measured using scanning electron microscopy (JEOL, JSM-7800F Prime, Field emission SEM, Japan). This is shown in Fig. 4. The performance of the fabricated nanopipettes was tested by injection of a solution containing 10 μM PE-labeled rhodamine B (PE-RB) into single chromaffin cells under a fluorescence microscope. Fig. S2† shows the steps of the penetration of the nanopipette (Fig. S2(B)†) and injection of the PE-RB solution into the cell (Fig. S2(C)†), as well as ejection of the nanopipette out of the cell (Fig. S2(D)†) without damaging the cell membrane and its structure. Nanopipettes were then filled with a solution containing a type of phospholipid or a blank solution using a Hamilton syringe and were used for the intracellular lipid supplementation. Fig. S3(A) and (B)† illustrate a representative cell during and after phospholipid injection, respectively.

A grid micro-pattern slide (SL5 cell finder, JD Photo Data, UK) was attached underneath the culture dish and used to address each cell during the injection. The treated cells were mapped and relocated by reading the coordinates of the cell finder grid under a microscope eyepiece for the following amperometric measurements. Nanopipettes were fixed above the inverted microscope by a holder (Axon Instruments, Union City, CA) and then connected to a microinjector system (Femtojet, Eppendorf, Germany). The injection parameters including injection time and pressure were tested over a recommended range specified for gentle injection into the single cell provided by Femtojet guideline.^27^ To avoid inflation or bursting the cells, the phospholipids were eventually injected at 0.2 s injection time and 100 hPa injection pressure. Although femtoliter-range volume of the phospholipid solution is injected by Femtojet into the cells; however, a slight change in the relative amount of membrane phospholipid are adequate to induce significant effects on the exocytosis.^28^ Two different micromanipulators (Thorlabs Inc. Newton, NJ) for the coarse and fine control of the nanopipette positioning were used. After injection, the cells were maintained in a humidified incubator at 37 °C, 5% CO_2_ for 30 min. A cell viability test, verified by the trypan-blue exclusion method, was carried out to show that the cell viability is not affected by insertion of 500 nm pipette (thanks to the short injection time, 0.2 s) and incubation of 0.1% DMSO.

A more detailed Experimental overview is given in the ESI.†

## Conflicts of interest

The authors declare no competing financial interest.

## Supplementary Material

SC-011-D0SC03683H-s001
