# Supplementary material for: Intracellular injection of phospholipids directly alters exocytosis and the fraction of chemical release in chromaffin cells as measured by nano-electrochemistry
Source: Chem Sci. 2020 Oct 6;11(43):11869–76. doi: 10.1039/d0sc03683h (PMC8162797; doi:10.1039/d0sc03683h)
Supplement: SC-011-D0SC03683H-s001 [file SC-011-D0SC03683H-s001.pdf]

Electronic Supplementary Material (ESI) for Chemical Science.
This journal is © The Royal Society of Chemistry 2020

Electronic Supplementary Information

Intracellular injection of phospholipids directly alters exocytosis and the fraction of chemical release in chromaffin cells as measured by nano-electrochemistry

Mohaddeseh Aref[‡], Elias Ranjbari[‡], Armaghan Romiani, Andrew Ewing[*]
Department of Chemistry and Molecular Biology, University of Gothenburg, Gothenburg, Sweden

[‡]Equal contribution to the work
[*]Corresponding author
E-mail: andrew.ewing@chem.gu.se

Results and discussion

Detailed Explanation of Table 1

Table 1 compares the difference in the trends of exocytotic parameters in previous work and the present work. Uchiyama et al.,[1] observed a faster dynamic in closing the exocytotic pore after extracellular incubation of the PC12 cells with PE which is observable as a depression in the t_{half} of the spikes. In contrast, we observed an elevation in the t_{rise}, t_{half}, and t_{fall} that shows slower dynamics of the pore for the intracellular incubation of Chromaffin cells with PE. While Uchiyama et al. observed a depression in the catecholamine released by the extracellularly incubated cells with PE, we observed an enhancement in the number of released molecules following intracellular incubation of the cells with PE. Furthermore, the trend that was observed in the dynamics of exocytosis affected by incubation of cells with PC in the paper published by Uchiyama et al. is opposite to that observed in our present work as a result of intracellular incubation of cells with PC. They reported slower dynamics in pore opening while we observed a shorter time that the pore remains open. The trend that we observed after nano-injection of the LPC was also different to that observed in Amatore et al.[2] Their results demonstrated that localisation of LPC in the outer leaflet favours the catecholamine release and increases the I_{max} and N. In our work, the injection of LPC into the cell cytoplasm to affect the inner leaflet of the cell membrane and outer leaflet of the vesicles caused an unfavourable curvature for exocytotic release and diminished the I_{max} and N. The comparison of the effects of intra- and extra-cellular LPC reveals that the fusion pore and so the efflux of neurotransmitter is larger when LPC is incorporated in the outer leaflet of the cell membrane.

Results of pre-spike parameters

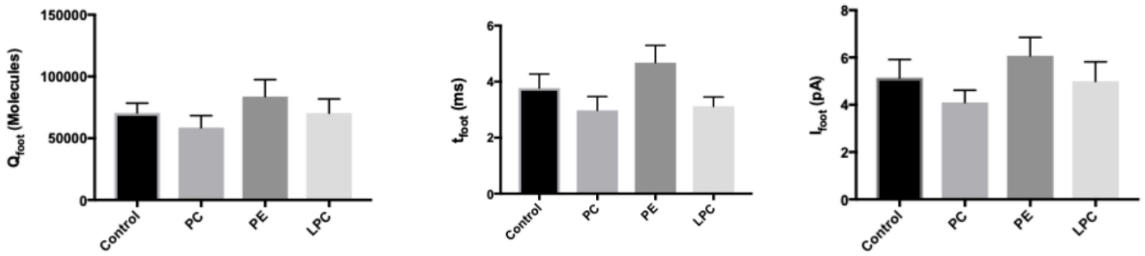

Figure S1. Effects of intracellular phospholipids on pre-spike parameters. Bar plots from exocytosis experiments measured by SCA for PC, PE, and LPC. The number of catecholamine molecules released, foot duration and foot current. Data show mean of median ± SEM. The data shows no significant difference with 95% confidence interval.

Additional Experimental

Chemicals and Solutions

All reagents were obtained from Sigma-Aldrich, unless otherwise stated. Stock solutions of 200 µL PC, PE, LPC were prepared in PBS containing 0.1% DMSO at the day of experiment freshly. Also, a blank PBS solution just containing 0.1% DMSO was prepared and used for injection as control cells. So, the control cells also exposed to injection to have the same condition with the other treated cells. Locke's stock buffer contained NaCl (1540 mM), KCl (56 mM), $NaHCO_3$ (36 mM), glucose (56 mM), HEPES (50 mM), and 1% (v/v) penicillin, pH 7.4. This stock solution was diluted 10 times the day before the experiment and used for gland storage and rinsing the adrenal gland vein. Isotonic solution: NaCl (150 mM), KCl (5.0 mM), $MgCl_2{\cdot}6H_2O$ (1.2 mM), $CaCl_2$ (2.0 mM), glucose (5.0 mM), HEPES (10 mM), pH 7.4. Stimulation solution: NaCl (55 mM), KCl (100 mM), $MgCl_2{\cdot}6H_2O$ (1.2 mM), $CaCl_2$ (2.0 mM), glucose (5.0 mM), HEPES (10 mM), pH 7.4. Collagenase P (from Clostridium

histolyticum) was obtained from Roche, Sweden. Cell culture medium: DMEM/Ham's F12 1:1 supplemented with 10% (v/v) fetal bovine serum, 1% (v/v) penicillin, 1% (v/v) cytosine β-D-arabinofuranoside, 0.1% (v/v) 5-fuoro-2'-deoxyuridine. Homogenizing buffer: sucrose (230 mM), EDTA (1 mM), MgSO4 (1 mM), HEPES (10 mM), KCl (10 mM), cOmplete™ enzyme inhibitor (Roche, Sweden), DNase I (10μg/mL) (Roche), oligomycin (1 mM), pH 7.4, ~310 mOsm. All solutions were prepared by diluting the chemicals in 18 MΩ cm^{-1} water from a Purelab Classic purification system (ELGA, Sweden), and were filtered (0.2 µm) prior to use.

Chromaffin cell and vesicle isolation

Bovine adrenal glands were obtained from a local slaughterhouse and transported in a cold Locke's buffer. The chromaffin cells were isolated as previously described.[3] Briefly, after sterilizing the glands with a solution of 70% (v/v) ethanol:water, fat and connective tissue were trimmed away. Blood cells were cleared by rinsing the veins with Locke's buffer. The medulla was isolated after digesting the gland with collagenase P (0.2%, Roche, Sweden) treatment. Digested medulla was filtered over steel sieves and diluted with Locke's buffer to reduce the activity of collagenase P. The cells were pelleted at 300g for 10 min at room temperature, and the pellet obtained was resuspended in Locke's buffer and filtered over a sterile 100 µm nylon mesh. The chromaffin cell suspension was mixed with sterile Percoll/Locke's buffer (10:1) and centrifuged at 18600g (Avanti J-20XP, Beckman Coulter, IN) for 20 min at room temperature. The top layer of the density gradient was collected and filtered over 100 µm nylon mesh. Again, the cell suspension was diluted with Locke's buffer and centrifuged at 300g for 10 min at room temperature to exclude Percoll. About 5×10^5 cells were seeded on 60 mm collagen (IV) coated plastic dishes (Corning Biocoat, VWR, Sweden) and maintained in a humidified incubator at 37 °C, 5% CO_2 prior to experiments. For vesicle isolation, a protocol developed by the R. Borges group was used.[4] Briefly, the medulla was mechanically homogenized in homogenizing buffer, and the vesicles were purified using a series of centrifugation steps: 1000g for 10 min to remove whole cells followed by 10000g to pellet vesicles. All centrifugation was performed at 4°C. The final pellet of vesicles was resuspended and diluted in homogenizing buffer and subsequently used for electrochemical measurements.

Carbon fiber micro-disk electrode

The Carbon fiber micro-disc electrode was fabricated as previously described.[5] A carbon fiber with 33-µm diameter was aspirated into a borosilicate capillary (1.2 mm O.D., 0.69 mm I.D., Sutter Instrument Co., Novato, CA, U.S.A.). Then, the capillaries were pulled using a micropipette puller (Model P-1000, Sutter Instruments Co., Novato, CA, U.S.A.) and the carbon fiber was cut at the glass junction. The gap between the carbon fiber and glass was sealed by dipping the pulled tip in a solution of epoxy (Epoxy Technology, Billerica, MA, U.S.A) to fixate the fiber. The glued electrodes were rested in an oven at 100°C overnight to complete the sealing step. The sealed electrodes were beveled at 45° angle (EG-400, Narishige Inc., London, UK). Prior to use the electrode for the main experiments the electrode response was tested by performing cyclic voltammetry (-0.2 to 0.8 V vs. Ag|AgCl, at 100 mV/s) in solution of dopamine (100 µM, in PBS, pH 7.4). Only electrodes showing good reaction kinetics and stable steady-state currents that were in agreement with theoretically calculated values for 33-µm disc electrodes were used for the experiments.

Amperometric experiments

Prior to each amperometric (SCA and IVIEC) experiment, cell cultured medium was removed from the culture dish and the cells were rinsed with pre-warmed isotonic solution. Cells were kept at 37°C on the microscope stage during the entire experiment. Electrochemical recordings from single chromaffin cells were performed on an inverted microscope (IX81, Olympus, Japan), in a Faraday cage. The working electrode was held at +700 mV versus a home-made Ag/AgCl reference electrode using an Axopatch 200B potentiostat (Molecular Devices, Sunnyvale, CA). Release was stimulated (5 s, 20 psi pulses

(Picospritzer II, General Valve Corporation, Fairfield, NJ)) with a high K^+ solution. Control and phospholipid-injected cells were assayed successively on the same day. SCA and IVIEC were alternatively performed in the same dishes.
The VIEC experiment of individual vesicular content was performed by applying a constant potential of +700 mV (vs. Ag/AgCl) to the working electrode. For vesicle treatments, a portion of the vesicles was carefully diluted with the different lipid solutions (PC, PE, and LPC in 0.1% DMSO:homogenizing solution) and blank solution (0.1% DMSO:homogenizing solution) as control experiment. The obtained suspensions were kept at room temperature for 30 min before pelleting down the vesicles to exclude the DMSO and finally resuspend in pure homogenizing solution for following electrochemical analysis. The disc electrodes were dipped in vesicle suspensions including the control, PC, PE, and LPC for 20 min at room temperature and then recorded in homogenizing buffer for 20 min at 37 °C.

Data Acquisition and Analysis

The signal output was filtered at 2 kHz using a 4-pole Bessel filter and digitized at 10 kHz using a Digidata1440A (Molecular Devices). The data were processed in IgorPro (Wavemetrics, Lake Oswego, OR).[6] A binomial filter was set to 1 kHz, and the detection limit was set to five times the RMS noise (0.7-1.0 pA) measured from the initial baseline in each measurement. In addition, traces were manually checked for potential false detections done by the software. Spike characteristics were determined as number of molecules based on the charge measured in each spike, t_{rise}= time from 25 to 75% of maximum during the increase of the spike, t_{fall} = time from 75 to 25% of maximum during the decrease of the spike, $t_{1/2}$ = full spike width at half-maximum, t_{foot} = duration of pre-spike foot from initial increase from baseline to the start of the spike, number of molecules foot = calculated from the charge measured in the foot. The medians from all cells were pooled, and groups were statistically analyzed using Prism 7 (GraphPad, La Jolla, CA) with an unpaired two-tailed Mann−Whitney rank sum test.

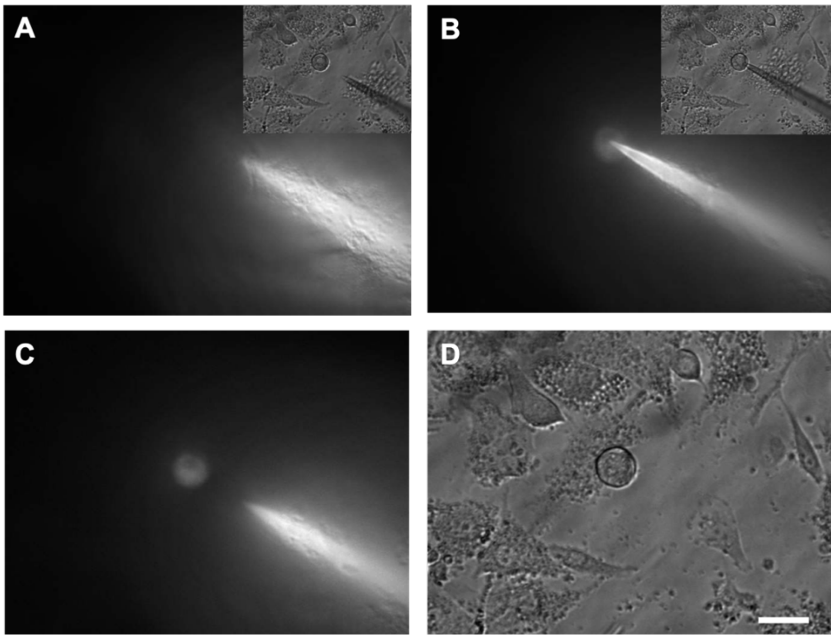

Figure S2. Performance of injection process using fabricated nanopipette into a single chromaffin cell. RB-PE used for injection as a fluorescent dye. Fluorescent images: (A) Before injection, (B) during injection, (C) after injection. Insets in A and B are the relevant bright field images. (D) Bright field image of the same single cell after injection. The scale bar is 20 μm.

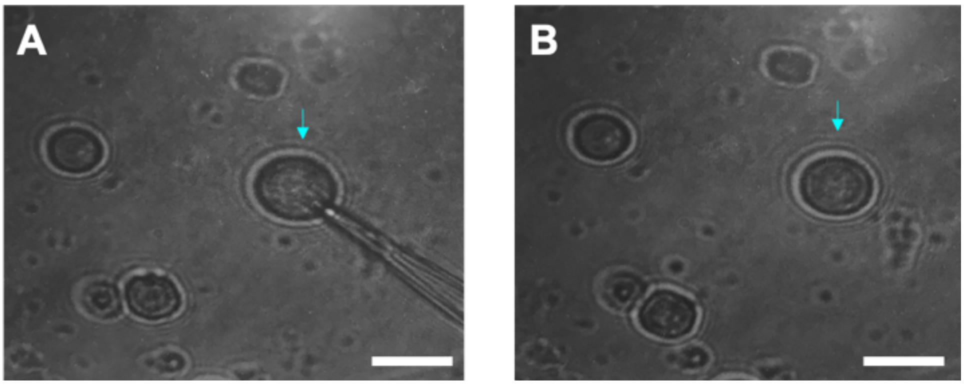

Figure S3. Bright field images of the chromaffin single cell during injection (A) and after injection to the same cell (B). The scale bar is 20 μm.

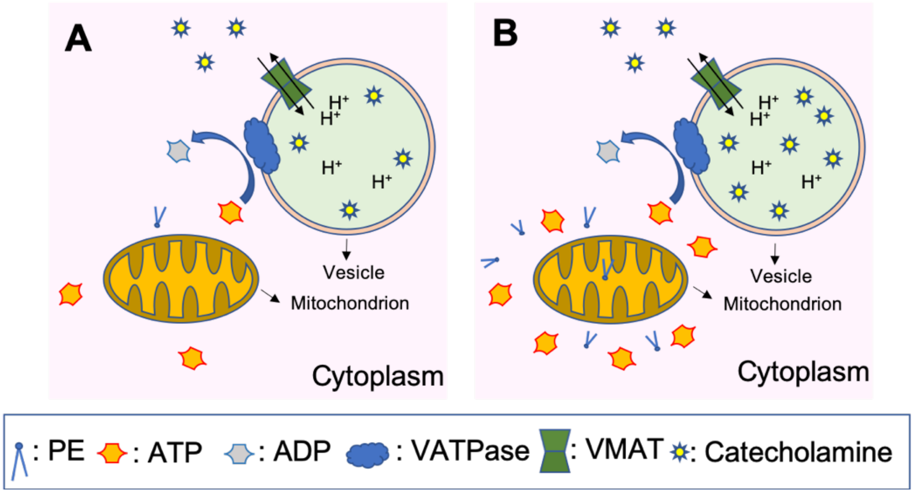

Scheme S1. Scheme for the proposed mechanism of vesicular storage alteration following PE intracellular supplementation.

References

1. Uchiyama, Y.; Maxson, M. M.; Sawada, T.; Nakano, A.; Ewing, A. G., Phospholipid mediated plasticity in exocytosis observed in PC12 cells. *Brain research* **2007,** *1151*, 46-54.
2. Amatore, C.; Arbault, S.; Bouret, Y.; Guille, M.; Lemaître, F.; Verchier, Y., Regulation of exocytosis in chromaffin cells by trans-insertion of lysophosphatidylcholine and arachidonic acid into the outer leaflet of the cell membrane. *ChemBioChem* **2006,** *7* (12), 1998-2003.
3. T O'Connor, D.; Mahata, S. K.; Mahata, M.; Jiang, Q.; Hook, V. Y.; Taupenot, L., Primary culture of bovine chromaffin cells. *Nature protocols* **2007,** *2* (5), 1248-1253.
4. Pardo, M. R.; Estévez-Herrera, J.; Castañeyra, L.; Borges, R.; Machado, J. D., Isolation of mouse chromaffin secretory vesicles and their division into 12 fractions. *Analytical biochemistry* **2017,** *536*, 1-7.

5. Li, X.; Dunevall, J.; Ewing, A. G., Using Single-Cell Amperometry To Reveal How Cisplatin Treatment Modulates the Release of Catecholamine Transmitters during Exocytosis. *Angewandte Chemie* **2016,** *128* (31), 9187-9190.
6. Mosharov, E. V.; Sulzer, D., Analysis of exocytotic events recorded by amperometry. *Nature methods* **2005,** *2* (9), 651-658.
